# Red light promotes sea slug growth while blue light increases longevity of stolen chloroplasts

**DOI:** 10.1242/bio.062714

**Published:** 2026-09-28

**Authors:** Carlos Paya, Heta Mattila, Vesa Havurinne, Felisa Rey, Sónia Cruz, Paulo Cartaxana

**Affiliations:** ^1^ Centre for Environmental and Marine Studies (CESAM), Department of Chemistry, University of Aveiro, 3810-193 Aveiro, Portugal; ^2^ CESAM, Department of Biology, University of Aveiro, 3810-193 Aveiro, Portugal; ^3^ Molecular Plant Biology unit, Department of Life Technologies, University of Turku, 20014 Turun Yliopisto, Finland; ^4^ Mass Spectrometry Centre and Associated Laboratory for Green Chemistry of the Network of Chemistry and Technology (LAQV-REQUIMTE), Department of Chemistry, University of Aveiro, 3810-193 Aveiro, Portugal

**Keywords:** Kleptoplast, Light spectrum, Photosynthesis, Sacoglossa, Starvation, Water depth

## Abstract

In Metazoa, long-term sequestration of photosynthetically active algal chloroplasts – kleptoplasts – is a distinctive physiological trait of sacoglossan sea slugs. In this study, we examined the effects of the light spectrum on the body weight and photosynthetic activity of the sea slug *Elysia crispata*. Laboratory-reared specimens were exposed to equivalent irradiances of white, red, or blue light during feeding and starvation experiments. Sea slugs fed under red light increased their weight at a faster pace than specimens fed under white or blue light. At the end of the feeding trial, chlorophyll *a* concentration – a proxy for kleptoplast abundance – was significantly higher under blue light. Progressive weight loss was observed in starved sea slugs under all light spectra, but an initial transient weight increase was observed under red light. Photosystem II activity and photosynthetic electron transport rate declined in starvation, with the decrease being fastest under red light and slowest under blue light. Results suggest a trade-off between increased photosynthetic rates and kleptoplast degradation, leading to increased growth (under red light) and enhanced kleptoplast longevity, possibly due to reduced photooxidative damage (under blue light). We hypothesise that the higher degree of parapodia closure observed under blue light shields kleptoplasts from light-induced damage, effectively extending their lifespan.

## Teaser

**Summary:** In photosynthetic sea slugs, red light promotes growth, while blue light prolongs the functionality of stolen algal chloroplasts.

## INTRODUCTION

Kleptoplasty is the ability of a host organism to sequester and maintain functional chloroplasts from prey algae (Rumpho et al., 2011; Pierce and Curtis, 2012). In the animal kingdom, this phenomenon is found almost exclusively in sacoglossan sea slugs, often termed ‘leaves that crawl’ or ‘solar-powered’ sea slugs (Trench, 1975; Rumpho et al., 2000). The ‘stolen’ chloroplasts were first termed kleptoplasts by Waugh and Clark (1986) in analogy to the retention of ingested nematocysts by nudibranchs (kleptocnidae; Hadzi, 1963). Several studies, using either inorganic labelled ^14^C or ^13^C, indicate that photosynthesis-derived metabolites are translocated from the kleptoplasts in the digestive tubules of the sea slugs into kleptoplast-free tissues (e.g. Trench et al., 1973; Cartaxana et al., 2021). Kleptoplast photosynthesis has been shown to be relevant in minimising weight loss and increasing survival during periods of food scarcity in several sacoglossan sea slugs (e.g. Hinde and Smith, 1972; Yamamoto et al., 2013; Cartaxana et al., 2017). In addition, sea slugs may obtain nutritive benefits under prolonged starvation by targeting kleptoplasts for degradation and using their starch reserves (Laetz et al., 2017).

The longevity of kleptoplasts varies considerably across sacoglossan sea slugs, ranging from only a few days to several months (Clark et al., 1990; Evertsen et al., 2007). *Elysia crispata* is a long-term-retention sea slug capable of maintaining functional kleptoplasts for over 1 month from a variety of macroalgal prey (Middlebrooks et al., 2019). Curtis et al. (2015) compared kleptoplast longevity in *E. crispata* when fed exclusively on green alga *Bryopsis plumosa* or *Penicillus lamourouxii* and found that chloroplasts from *P. lamourouxii* were retained for 4 weeks longer than those from *B. plumosa*. *Elysia timida* and its closely related sister species *Elysia cornigera* illustrate the contrast between long-term and short-term retention: although both feed exclusively on the alga *Acetabularia acetabulum*, only *E. timida* is able to maintain functional chloroplasts over extended periods (de Vries et al., 2015; Laetz and Wägele, 2018). Hence, kleptoplast longevity appears to be dependent on the intrinsic robustness of the original chloroplasts as well as on species-specific characteristics of animal cells (Cruz and Cartaxana, 2022).

Kleptoplasts do not divide once in host cells, and many of the proteins required for the functioning and maintenance of the photosynthetic apparatus are nucleus encoded (Ponce-Toledo et al., 2019; Sibbald and Archibald, 2020). Therefore, kleptoplasts undergo gradual degradation after acquisition by the sea slug cells. Apart from the algal donor/sea slug combination, the rate and pattern of this degradation depend significantly on abiotic factors such as light and temperature (Vieira et al., 2009; Rey et al., 2023; Laetz et al., 2024). Light intensity, in particular, has been shown to play a key role in determining kleptoplast longevity, with high irradiance accelerating kleptoplast degradation (Vieira et al., 2009; Klochkova et al., 2013; Donohoo et al., 2020). A less explored but potentially important variable is the spectral composition of light and its impact on kleptoplast functionality. *E. crispata* occurs from surface waters to depths of up to 25 m (Krug et al., 2016; Vital et al., 2021, 2023), and the spectral composition of available light changes within this depth range due to differential wavelength attenuation, with blue wavelengths penetrating deeper into the water column compared to red wavelengths. In this study, we examined the effects of light spectra (white, red, and blue) on kleptoplast photosynthesis and longevity in this tropical photosynthetic sacoglossan sea slug during feeding and starvation conditions in the laboratory.

## RESULTS

### Effects of the light spectrum during feeding

Young *E. crispata* juveniles fed once per week (‘feeding experiment’) increased their fresh weight over the 42-day experimental period under the three light spectral treatments (white, red, and blue) (Fig. 1; Dataset 1). After 14 days, sea slugs exposed to red light displayed higher average fresh weights, although at that point, the differences were not statistically significant compared to sea slugs fed under white and blue light. This trend intensified over time, and the first significant difference emerged on day 21 between sea slugs under red and blue light (*P*<0.01). By day 28, sea slugs under red light exhibited significantly higher fresh weights than those under both blue and white light (*P*<0.01 for both comparisons), and these differences were maintained until the end of the experiment.

**Fig. 1.**
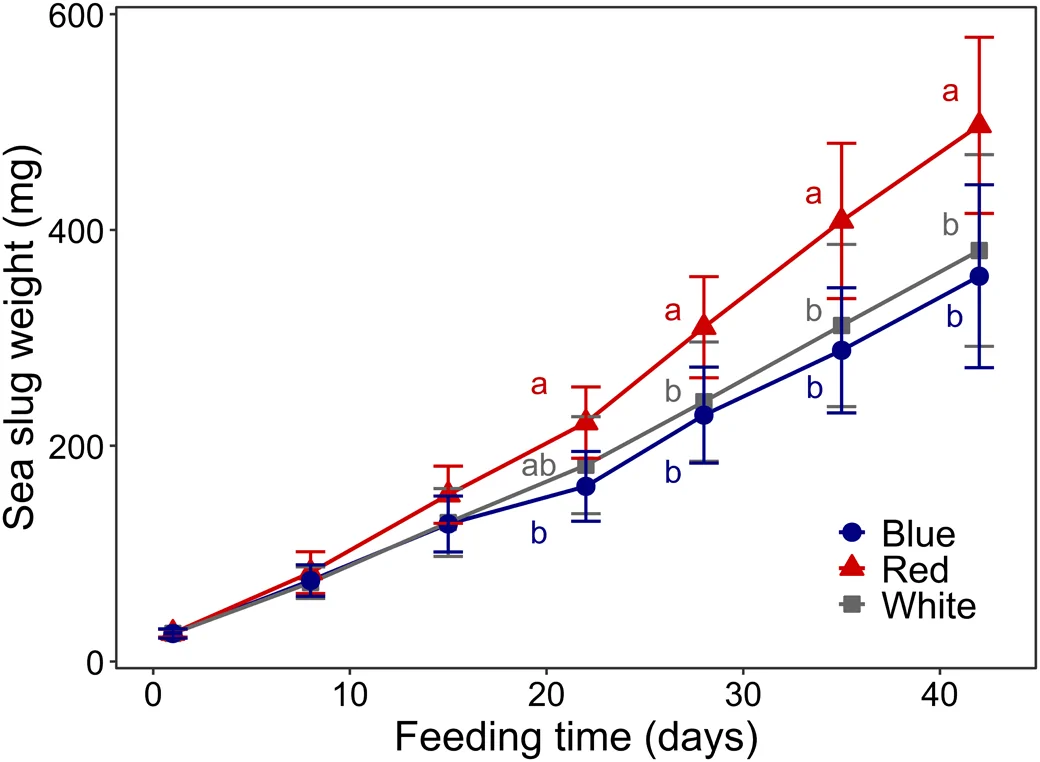
**Fresh weight of *E. crispata* reared under different light spectra: white, red, and blue.** Sea slugs were maintained at a PPFD of 60 μmol photons m^−2^ s^−1^ and fed once per week with the macroalga *Bryopsis* sp. Data are means±s.d. of ten individuals. Different letters indicate significant differences between light treatments for each feeding time (*P*<0.05).

Photosystem II (PSII) activity, estimated through the chlorophyll *a* (Chl *a*) fluorescence parameter Fv/Fm, remained relatively stable at around 0.65, indicating that feeding once per week for 48 h was sufficient to maintain high photosynthetic efficiency (Fig. 2A). Although major differences were not identified between light spectra, an overall decreasing Fv/Fm trend was observed under red light. Photosynthetic electron transfer rates, estimated through the Chl *a* fluorescence parameter rETRmax (maximum relative electron transport rate), decreased in all treatments after 21 days (Fig. 2B). By the end of the 42-day experiment, rETRmax values increased in all treatments, returning to levels comparable to those measured at the beginning of the feeding experiment. No statistically significant differences in rETRmax were detected among the light spectrum treatments.

**Fig. 2.**
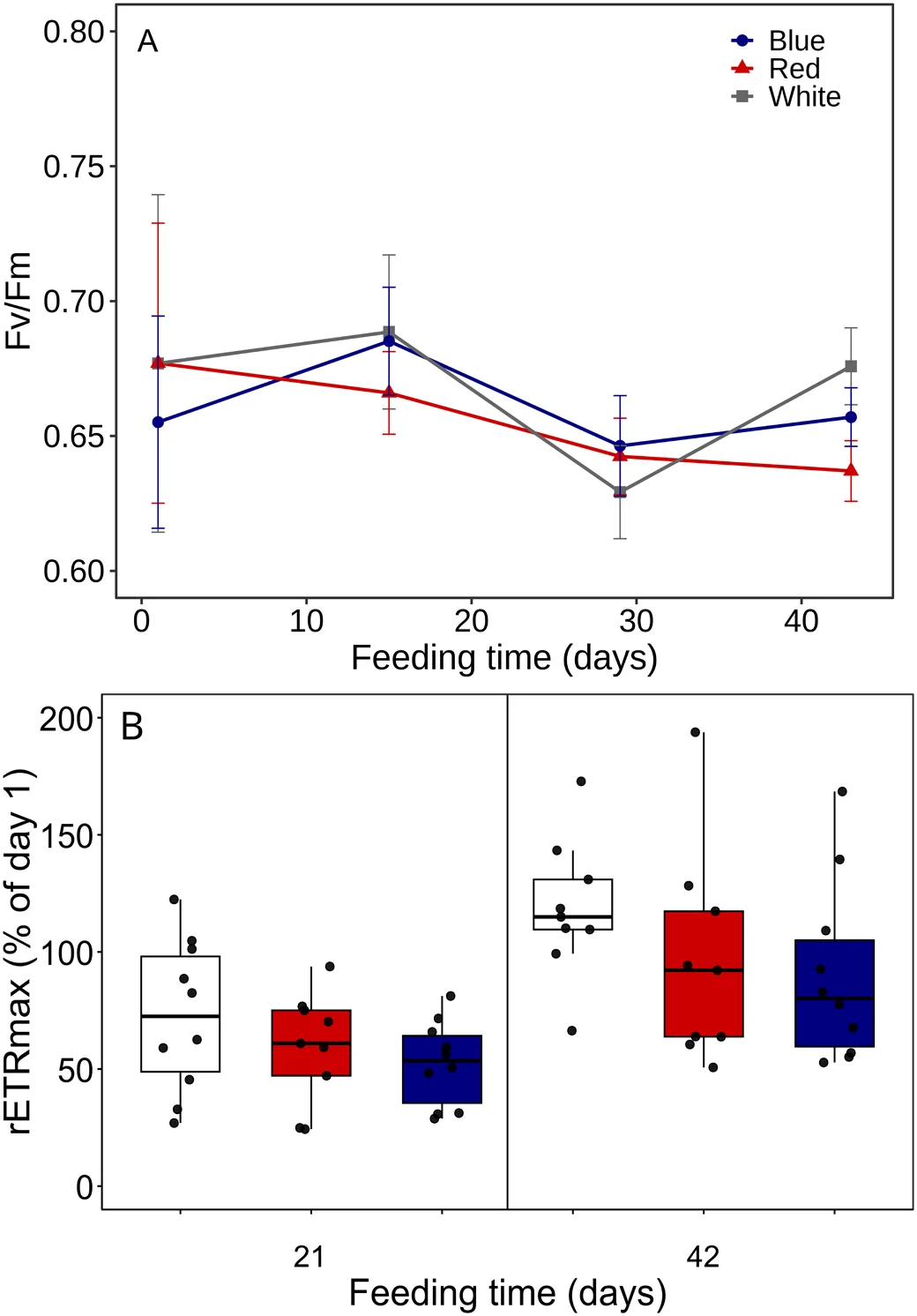
**Photosynthesis of *E. crispata* kleptoplasts during feeding under different light spectra.** (A) PSII activity (Fv/Fm) and (B) rETRmax in *E. crispata* reared under different light spectra: white, red, and blue. Sea slugs were maintained at a PPFD of 60 μmol photons m^−2^ s^−1^ and fed once per week with the macroalga *Bryopsis* sp. In panel A, data are means±s.d. of ten individuals. In panel B, the line shows the median; the top and bottom of the box are the 75th and 25th percentiles, respectively; and the whiskers represent the non-outlier maximum and minimum values of ten individuals. Points indicate the individual measurement values.

Analysis of Chl *a* content in sea slugs revealed statistically significant differences (*P*<0.05) among the light treatments (Fig. 3). At the end of the experimental period, Chl *a* content was significantly higher under blue light than under red or white light (in both cases, *P*<0.05). Confocal microscopy images revealed no clearly visible differences in kleptoplast morphology or distribution across light treatments (Fig. S2).

**Fig. 3.**
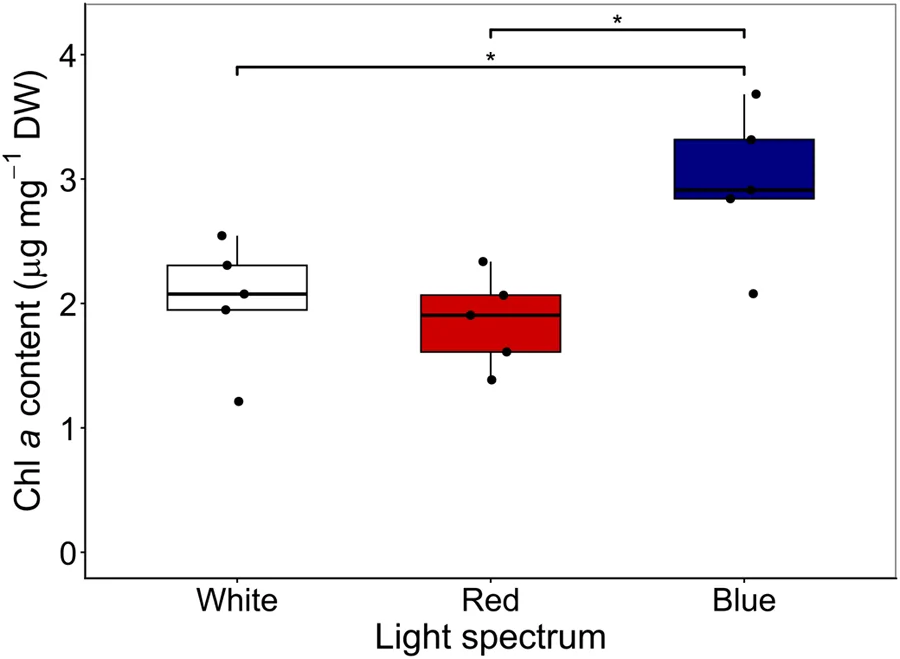
**Chl *a* content of *E. crispata* reared for 42 days under different light spectra: white, red, and blue.** Sea slugs were maintained at a PPFD of 60 μmol photons m^−2^ s^−1^ and fed once per week with the macroalga *Bryopsis* sp. The line shows the median; the top and bottom of the box are the 75th and 25th percentiles, respectively; and the whiskers represent the non-outlier maximum and minimum values of five individuals. Points indicate the individual measurement values. Asterisks mark significant differences between the indicated treatment groups (*P*<0.05).

### Effects of light spectrum under starvation

Adults of *E. crispata* were maintained under white, blue, and red light without a food source (‘starvation experiment’). Sea slug fresh weight showed a progressive decline in all treatments, with specimens reaching approximately 50% of their original weight after 42 days of starvation (Fig. 4). However, during the first days of starvation, differences were observed among treatments: sea slugs under red light showed a marked increase in fresh weight, which was not observed under white or blue light. After 14 days of starvation, sea slugs' relative fresh weight under red light was significantly higher than that under blue and white light (*P*<0.001 and *P*<0.05, respectively). These differences persisted for two additional weeks between blue and red light, but from day 35 onwards, no significant differences in fresh weight were observed between any of the light treatments.

**Fig. 4.**
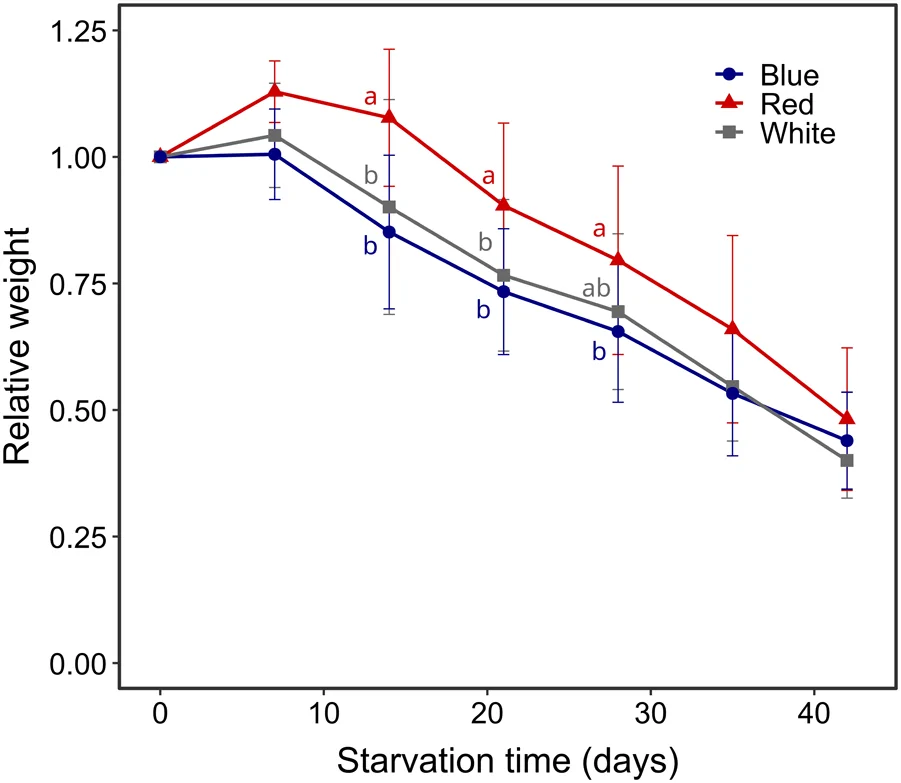
**Fresh weight of *E. crispata* starved under different light spectra: white, red, and blue.** Sea slugs were maintained at a PPFD of 60 μmol photons m^−2^ s^−1^. Data are means±s.d. of ten individuals. Different letters indicate significant differences between light treatments for each starvation time (*P*<0.05).

In terms of PSII activity (Fv/Fm), sea slugs under red light exhibited a sharper average decrease over the course of the starvation experiment compared to those under blue light, while sea slugs under the white light treatment showed an intermediate decline (Fig. 5A). After 14 days of starvation, Fv/Fm under blue light was significantly higher than that under red light (*P*<0.05). By day 21 of starvation, significant differences were found among all treatments (in all cases, *P*<0.05). These differences persisted throughout the experimental period, with sea slugs under red and white light showing undetectable Fv/Fm after 42 days of starvation. Relative ETRmax showed a decreasing trend consistent with Fv/Fm results (Fig. 5B). Differences between light treatments were observed after 14 days of starvation, with statistically significantly higher rETRmax under blue light when compared to that under red light (*P*<0.05). By day 28 of starvation, all treatments differed significantly from each other, with sea slugs showing the highest rETRmax under blue light, intermediate values in white light, and the lowest in red light (in all cases, *P*<0.01). Survival under starvation, ranging from 43 to 115 days, did not differ significantly across the different light spectra (Fig. 6).

**Fig. 5.**
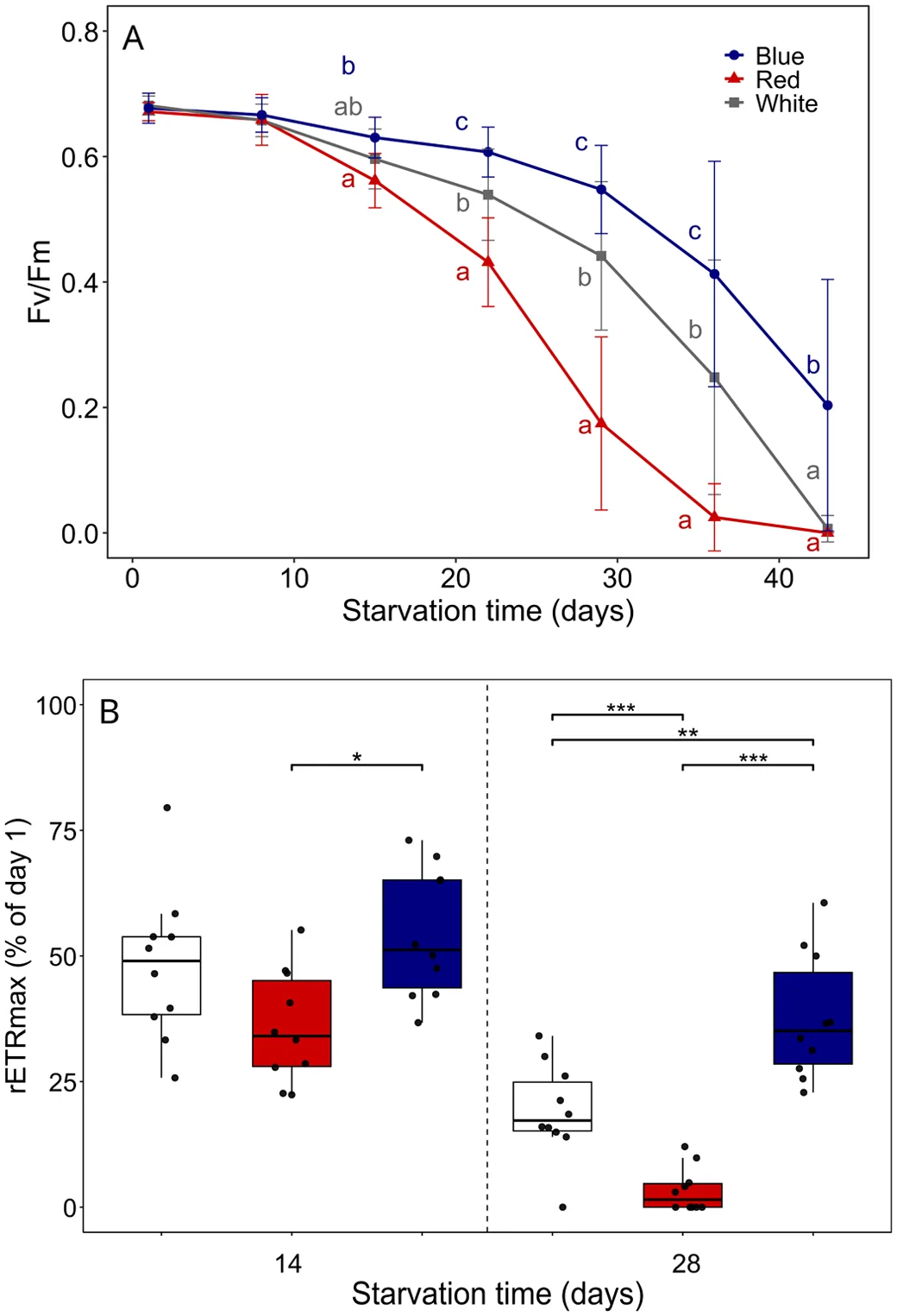
**Photosynthesis of *E. crispata* kleptoplasts during starvation under different light spectra.** (A) PSII activity (Fv/Fm) and (B) rETRmax in *E. crispata* reared under different light spectra: white, red, and blue. Sea slugs were maintained at a PPFD of 60 μmol photons m^−2^ s^−1^. In panel A, data are means±s.d. of ten individuals. Different letters indicate significant differences between light treatments for each starvation time (*P*<0.05). In panel B, the line shows the median; the top and bottom of the box are the 75th and 25th percentiles, respectively; and the whiskers represent the non-outlier maximum and minimum values (*n*=10). Points indicate the individual measurement values. Asterisks mark significant differences between the indicated treatment groups (*P<0.05, **P<0.01, ***P<0.001).

**Fig. 6.**
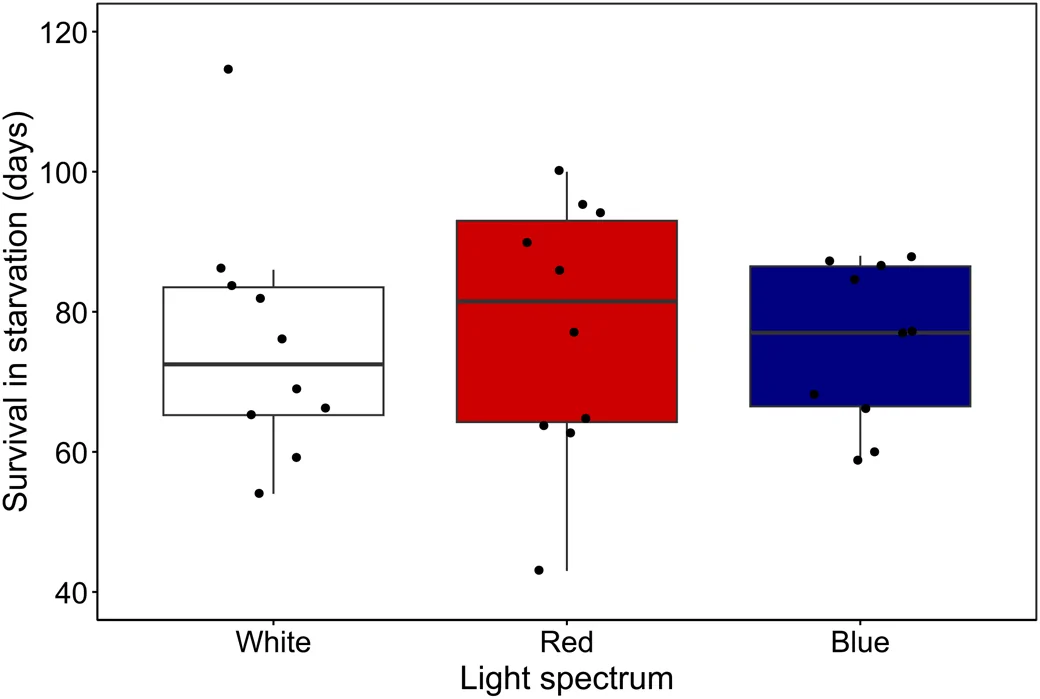
**Survival of *E. crispata* in starvation under different light spectra: white, red, and blue.** Sea slugs were maintained at a PPFD of 60 μmol photons m^−2^ s^−1^. The line shows the median; the top and bottom of the box are the 75th and 25th percentiles, respectively; and the whiskers represent the non-outlier maximum and minimum values of ten individuals. Points indicate the individual measurement values.

### Sea slug behaviour under different light spectra

When sea slugs, taken from growth conditions, were exposed to the different light spectra, the exposed dorsal area (EDA) was significantly lower under blue light than under white or red light (*P*<0.05 and *P*<0.001, respectively; Fig. 7), confirming the observation made during the feeding and starvation experiments that sea slugs closed their parapodia more pronouncedly under blue light. Furthermore, sequentially exposing animals to red and blue light confirmed this behaviour. When exposed to red light, sea slugs opened their parapodia, but a transition to blue light induced their closure (Movie 1). When light was switched back to red, sea slugs responded by opening their parapodia.

**Fig. 7.**
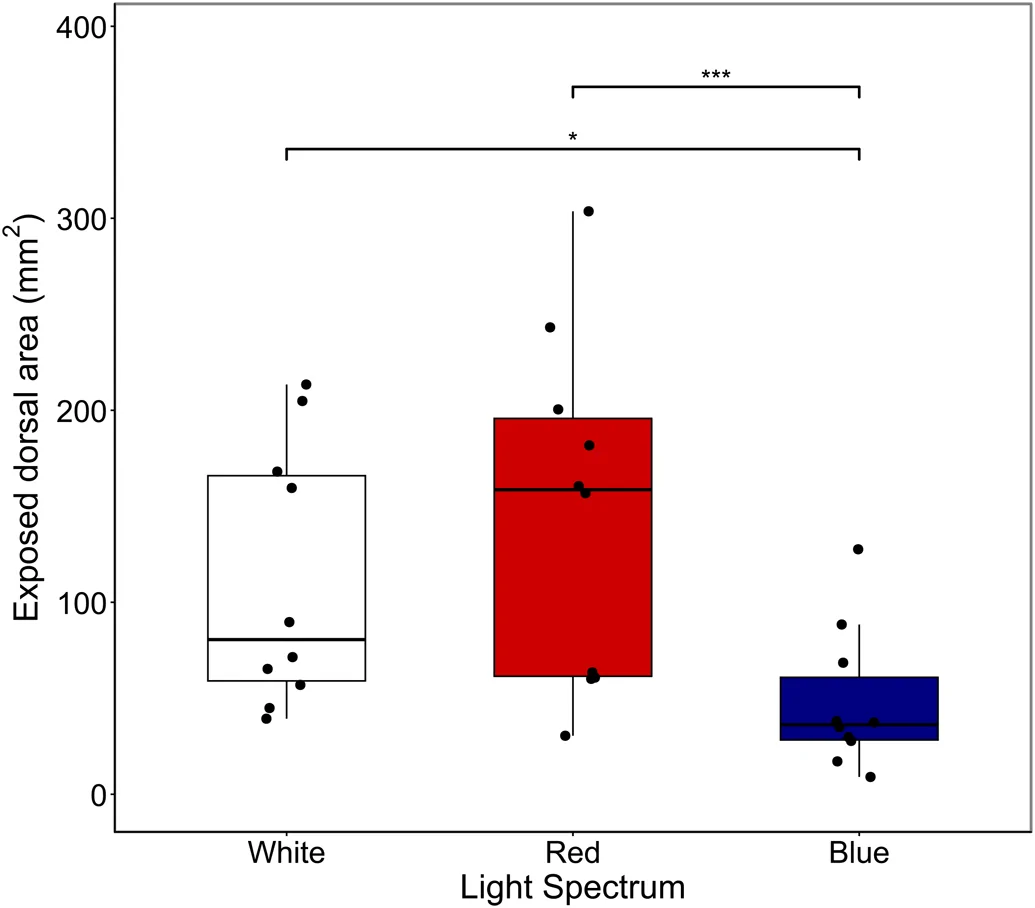
**EDA in *E. crispata* under different light spectra: white, red, and blue.** Sea slugs were exposed to a PPFD of 60 μmol photons m^−2^ s^−1^. The line shows the median; the top and bottom of the box are the 75th and 25th percentiles, respectively; and the whiskers represent the maximum and minimum values of ten individuals. Points indicate the individual measurement values. Asterisks mark significant differences between the indicated treatment groups (*P<0.05, ***P<0.001).

## DISCUSSION

### Red light promoted fresh weight gains, whereas blue light enhanced kleptoplast longevity

Juveniles of *E. crispata* exhibited sustained weight gain over time under all tested light spectra (white, red, and blue). No apparent differences in the feeding behaviour were observed, and the sea slugs emptied the cellular content of the provided food algae within 48 h, independently of the light treatment. However, a consistent trend of higher fresh weight under red light was observed. Hence, the pattern observed under red light may reflect (i) an increased contribution of metabolites derived from enhanced kleptoplast photosynthetic activity, (ii) mobilisation of internal reserves through kleptoplast degradation, or (iii) a synergistic contribution of both mechanisms.

Kleptoplastic sea slugs have been shown to incorporate inorganic carbon derived from kleptoplast photosynthesis in a variety of host molecules, such as sugars (Trench et al., 1973; Lopes et al., 2025), fatty acids (Cruz et al., 2020; Cartaxana et al., 2021), and polyketides (Ireland and Scheuer, 1979). High-resolution nanoscale secondary ion mass spectrometry isotopic imaging identified photosynthesis-derived carbon and nitrogen incorporation in kleptoplast-free organs such as the albumen gland and gonadal follicles (Cruz et al., 2020; Cartaxana et al., 2021). In addition to carbon fixation and translocation, kleptoplast-derived reserves may eventually be secured through the selective targeting and degradation of kleptoplasts. In the sea slug *E. timida*, some of the photosynthates produced in the kleptoplasts were not immediately transported to sea slug tissues but accumulated as starch grains for later use (Laetz et al., 2017). The authors suggested that kleptoplasts function as both nutritive producers and storage devices, helping sea slugs withstand extended periods of food shortage.

Recently, kleptoplasts in *E. crispata* were shown to be housed within host-derived organelles termed kleptosomes, which could fuse with lysosomes to degrade dysfunctional kleptoplasts (Allard et al., 2025). Sea slugs grown in the presence of food contained a limited pool of lysosomes, whereas starved *E. crispata* were enriched in lysosomes (Allard et al., 2025). This is in accordance with the findings of Laetz et al. (2017), reporting the mobilisation of starch reserves mainly during periods of prolonged starvation. Regardless, Allard et al. (2025) observed low but detectable rates of chloroplast digestion also in fed sea slugs, suggesting that the few observed lysosomes were capable of fusing with kleptosomes. In the present work, we report higher Chl *a* concentrations (relative to sea slug mass) in fed individuals under blue light than under red and white light. This result suggests that the rate of kleptoplast degradation is impacted by the light spectrum, with blue light enhancing kleptoplast longevity. Similarly, a study comparing the effects of blue, red, and combined blue and red light on the scleractinian coral *Stylophora pistillata* observed that red light alone or in combination with blue light reduced symbiont density (Wijgerde et al., 2014). A key question, therefore, is why kleptoplast degradation rates appear lower under blue light.

Studies on the light preferences of *E. crispata* have shown that this sea slug avoids red light (Weaver and Clark, 1981; Vital et al., 2026). Vital et al. (2026) hypothesised that *E. crispata* photoreceptors absorb mainly in the blue and green regions of the light spectrum and that sea slugs might perceive red light as extremely low light or the absence of light altogether. The absorption of blue light by the photoreceptors could therefore trigger an animal behavioural response that could shield the kleptoplasts, thus extending their lifespan. Indeed, photosynthetic sacoglossan sea slugs can fold their parapodia, potentially reducing the number of photons reaching the kleptoplasts (Cartaxana et al., 2019; Donà et al., 2022). In the present study, we show that sea slugs closed their parapodia more pronouncedly under blue light than under red or white light, leading to a significant reduction of the EDA. Furthermore, subjecting *E. crispata* to sudden changes of the light spectra revealed a consistent behaviour of parapodia closure under blue light and parapodia reopening under red light. Hence, it is possible that kleptoplasts in the sea slugs under blue light were, in fact, exposed to lower light levels due to the differential perception of light and consequent closure of the parapodia. This could lead to lower photosynthetic rates but also to less photooxidative stress and, subsequently, to slower stress-induced kleptoplast degradation under blue light.

Additionally, the apparent slower degradation of kleptoplasts under blue light may reflect a lower rate of photooxidative damage that could result from the preferential absorption of the blue photons near the tissue surface, whereas longer wavelengths are generally less attenuated and penetrate deeper into biological tissues (Ash et al., 2017). Consequently, red light may be more efficient in reaching kleptoplasts located deeper within the sea slug tissues, enhancing photodamage and turnover. Consistent with this interpretation, previous research on *E. timida* demonstrated that blue light (425–470 nm) induces damage to PSII at approximately half the rate observed under red light (660–690 nm), possibly due to absorption of shorter wavelengths by the sea slug tissue itself (Havurinne et al., 2022).

### In starvation, photosynthetic activity declined faster under red light than under blue light

In the starvation experiment, adult sea slugs showed a progressive loss of fresh weight across all treatments, indicating that kleptoplast photosynthesis alone is insufficient to maintain animal weight. Rates of fresh weight decrease in starving *E. crispata* are known to depend on the species of the macroalgal chloroplast donor, as well as on food abundance prior to the initiation of the starvation period (Curtis et al., 2015; Cartaxana et al., 2023). In our study, the early phase of the starvation experiment revealed distinct responses between light treatments: sea slugs under red light increased their weight during the first 2 weeks, suggesting that red light initially enhanced photosynthesis in the kleptoplasts in comparison with the other light spectra, supporting observations from the feeding trial. However, this advantage diminished over time, and by the end of the experimental period, all treatments converged towards similar weight losses.

Photosynthetic parameters Fv/Fm and rETRmax declined during starvation, indicating a progressive loss of kleptoplast functionality within the host sea slug cells. A comparable decrease on Fv/Fm has been previously reported for starved *E. crispata* pre-fed on *Bryopsis* spp. (Curtis et al., 2015; Cartaxana et al., 2023). Interestingly, sea slugs exposed to blue light maintained significantly higher Fv/Fm and rETRmax values during starvation compared to those exposed to red light, while those exposed to white light showed intermediate values. As previously discussed, increased light absorption by kleptoplasts, triggered by the opening of the parapodia under red light, could cause an initial boost of photosynthetic rates and carbon fixation. This could explain the initial weight gain under red light, but it could also lead to increased damage to kleptoplasts and, consequently, to the sharper decrease in Fv/Fm and rETRmax parameters, which, by the end of the experimental period, led to similar weight losses across all light treatments. On the other hand, blue light seems to induce parapodia closure, limiting photosynthesis but effectively shielding kleptoplasts from light-induced damage and, therefore, extending their lifespan in the animal cells. Despite the discussed effects on photosynthesis and kleptoplast longevity under starvation, survival did not differ significantly among light treatments.

Because kleptoplasts do not have the support of the algal nucleus for the repair of major components of the photosynthetic apparatus, increased light absorption under red light would lead to an earlier degradation of the photosynthetic machinery. The proposed increased kleptoplast photosynthetic rates and photosynthesis machinery impairment may also lead to earlier mobilisation of internal reserves through kleptoplast degradation, further contributing to the weight differences observed. Although we did not perform Chl *a* quantification on starved sea slugs because we were monitoring survival, visual observations suggest that sea slugs under red light bleached earlier than conspecifics under blue light (Fig. S3). Indeed, our data suggest that photosynthetic sea slugs can detect and target damaged kleptoplasts for degradation, possibly by monitoring their ATP production via the kleptosome's ATP-gated ion channels (Allard et al., 2025).

### Depth-related changes in light spectral composition and kleptoplasty in *E. crispata*

The sea slug *E. crispata* has been reported from the water surface up to a depth of 25 m (Krug et al., 2016; Vital et al., 2021, 2023). Across this depth range, the spectral composition of light changes significantly, with blue wavelengths penetrating deeper in the water column, whereas red light is preferentially absorbed at the upper water layers (e.g. Kirk, 1994). Hence, *E. crispata* may possess the capacity to adapt or acclimate along a light gradient ranging from high-irradiance, full-range light in shallower waters to low-irradiance, blue-dominated light in deeper waters. Our results highlight a complex interaction between the light spectrum and the maintenance of functional kleptoplasty in *E. crispata*. Exposure to red light seems to promote sea slug growth, possibly by transiently increasing photosynthetic rates and earlier kleptoplast degradation. On the other hand, blue light appears to promote closure of the parapodia, possibly leading to a lower rate of photooxidative damage, enhancing kleptoplast longevity. Changes in light spectral composition with depth can be a relevant environmental factor shaping kleptoplast functionality in *E. crispata*.

## MATERIALS AND METHODS

### *E. crispata* rearing

Sea slugs of the species *E. crispata*, originally collected in Florida, were reared in the laboratory for several generations (Cartaxana et al., 2026) and maintained in 340 l recirculating life support systems (LSS) using artificial seawater (ASW). The ASW was formulated using a commercial sea salt mixture (Red Sea Europe, Verneuil d'Avre et d'Iton, France) dissolved in reverse osmosis water (MO12000 E; Ambietel – Tecnologias Ambientais LDA, Matosinhos, Portugal). The systems were kept at 25°C and 35 parts per thousand (PPT) salinity. A 12-h:12-h light:dark photoperiod was established, with an irradiance of 60–80 μmol photons m^−2^ s^−1^ of photosynthetic photon flux density (PPFD) provided by ViparSpectra (V165, CA, USA) LED aquarium lights. The light intensity at the water surface was measured using a Spherical Micro Quantum Sensor and a ULM-500 Universal Light Meter (Walz, Effeltrich, Germany). Two trials were performed, hereafter referred to as the feeding and starvation experiments. For the feeding experiment, approximately 2-week-old juveniles were used, while for the starvation experiment, approximately 6-month-old adults were utilised. The animals used in the two experiments (feeding and starvation) were different specimens.

### *Bryopsis* sp. culturing

The macroalga *Bryopsis* sp. was cultivated in the laboratory to meet the dietary needs of *E. crispata*. This alga, originally obtained from the Kobe University Macroalgal Culture Collection in Japan (strain KU-0990), was grown in 2-l flasks using f/2 medium (without silica; Andersen et al., 2005), prepared in ASW, under constant aeration. The algal cultures were maintained at 20°C under a PPFD of 60–80 μmol photons m^−2^ s^−1^, delivered by LED lamps (Valoya 35 W, spectrum NS12, Finland).

### Sea slug feeding and starvation experiments

Floating trays containing wells (56 mm in diameter×60 mm in depth) were placed in LSS under the same temperature, salinity, photoperiod, and light intensity as described above. The bottoms of the wells were fitted with a mesh (0.5 mm for the feeding experiment and 2 mm for the starvation experiment) to allow water exchange. A powerhead (Turbelle nanostream 6015; Tunze, Penzberg, Germany) was installed to guarantee broad and diffuse water movement, enhancing water renewal inside the wells.

Sea slugs were randomly assigned to each light treatment, with ten individuals per light treatment. Three Reef Pulsar SPS-8 LED light sources (TMC, Hertfordshire, UK) were placed on top of three LSS. Each lighting rig and LSS was set for a different treatment: white, red, or blue light. This was achieved by using the following channels of the Reef Pulsar lights: channel 1 at 85% for the white light treatment; channel 6 at 99% for the red light treatment; and channel 3 at 97% for the blue light treatment. The spectra for the light treatments were measured using a LI-180 spectrometer (LI-COR, Lincoln, NE, USA; Fig. S1). The light intensity was set to 60 μmol photons m^−2^ s^−1^ for all three light treatments, which were conducted concurrently.

In the feeding experiment, individuals had initial fresh weights (mean±s.d.) of 26.1±3.9 mg (white light), 25.9±3.9 mg (red light), and 26.0±4.5 mg (blue light). Each animal received 100 mg of *Bryopsis* sp. every week for the first 2 weeks, followed by 150 mg from the third week onwards. No differential feeding behaviour was observed between spectral treatments. Colourless algal debris was removed from the wells after 48 h. Sea slug fresh weight and photosynthetic parameters (see below) were monitored for 42 days. At this time point, five fed sea slugs per treatment were used for confocal microscopy analysis, whereas the remaining five sea slugs were frozen in liquid nitrogen and kept at −80°C for pigment analysis (see below).

In the starvation experiment, sea slug initial weights (mean±s.d.) were 687.5±97.3 mg (white light), 676.7±121.7 mg (red light), and 634.8±73.0 mg (blue light). No food was provided during this experiment. Sea slug fresh weight and photosynthetic parameters (see below) were monitored for 42 days. However, sea slug survival was monitored until the last sea slug died. A sea slug was considered dead if not moving and not responsive when prodded in the rhinophores.

In both experiments, sea slugs were placed in separate wells, which prevented them from reproducing by cross-fertilisation. No egg masses were spawned during either of the experiments.

### Sea slug weight

The fresh weight of the sea slugs was monitored once a week throughout the experiments. Before being weighed, the sea slugs were placed for a few seconds in a mesh (200 μm), with absorbent paper underneath to remove excess water, and transferred to a weighing plate (PBA224I-1S; VWR, Portugal).

### Photosynthetic activity: Fv/Fm and relative ETRmax

The maximum quantum yield of PSII was estimated using an Imaging-PAM fluorometer Mini version (Walz, Effeltrich, Germany). This parameter was calculated as Fv/Fm=[Fm−Fo]/Fm, where Fo is the minimum fluorescence (only a weak measuring light on), and Fm is the maximum fluorescence (during a saturating light pulse). The Fo and Fm were measured after dark acclimation of sea slugs for a period of 30 min. Fluorescence images showing Fv/Fm were analysed with ImagingWin v2.56zg software by applying three areas of interest (AOIs) within the body of the specimens, avoiding the head and focusing on the dorsal body flaps (parapodia) where kleptoplasts are mainly distributed in the sea slug body. The ‘mean over AOI’ option was always enabled, which means that all pixel values over the whole AOI were averaged. The Fv/Fm of the sea slugs was monitored once every 2 weeks in the feeding experiment and every week in the starvation experiment.

Rapid light response curves (RLCs) were used to probe photosynthetic electron transfer rates by measuring chlorophyll fluorescence over a short sequence of increasing light intensities (Ralph and Gademann, 2005). In the RLC measurements, after specimens were acclimated to room light (PPFD of approximately 20 μmol photons m^−2^ s^−1^) for 5–15 min, individuals were immobilised by placing them on a black cloth (Havurinne et al., 2025). Samples were illuminated for 40 s with blue light at PPFDs of 1, 17, 39, 84, 178, 309 and 540 μmol photons m^−2^ s^−1^, and after each light intensity, a saturating light pulse was fired. The relative electron transport rate (*rETR*) through PSII was estimated using the following equation:

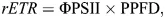
where ΦPSII is an estimate of the effective quantum yield of PSII photochemistry [(Fm′−F)/Fm′, where F and Fm′ are, respectively, the minimum and maximum fluorescence under the light conditions], and PPFD (μmol photons m^−2^ s^−1^) is the actinic light intensity of the RLC light steps generated by the internal LED lamp of the Imaging-PAM. Each RLC was modelled using the method of Eilers and Peeters (1988) to estimate the rETRmax. rETRmax values were expressed as a percentage relative to each individual's initial rETRmax measured at the beginning of the experiment. Measurements were performed at the beginning of the experimental period and after 3 and 6 weeks in the feeding experiment and after 2 and 4 weeks in the starvation experiment.

### Chl *a* quantification

Chl *a* analysis was conducted using high-performance liquid chromatography (HPLC), following the protocol outlined by Cruz et al. (2014). Sea slugs were initially freeze dried and homogenised, and approximately 4 mg of dry weight was extracted for 20 min in 95% cold buffered methanol (containing 2% ammonium acetate) at −20°C, after 1 min of sonication. The resulting extracts were filtered through 0.2 μm polytetrafluoroethylene (PTFE) membrane filters and then injected into a Prominence-i LC 2030C HPLC system (Shimadzu, Kyoto, Japan) equipped with a photodiode array detector. Chromatographic separation was performed using a Supelcosil C18 reverse-phase column (250×4.6 mm, 5 μm particle size; Sigma-Aldrich, St Louis, MO, USA). Chl *a* was identified based on retention time and absorption spectrum, and concentrations were calculated from the signals in the photodiode array detector. A calibration curve for Chl *a* was constructed with five dilutions of a pure standard from DHI (Hørsolm, Denmark).

### Confocal microscopy and image analysis

At the end of the feeding experiment, sea slug samples were prepared by quick decapitation with a razor blade and then by cutting a piece of parapodium from the midsection of the body. Afterwards, the cut pieces were imaged with a confocal microscope of a Mica Microhub (Leica Microsystems, Wetzlar, Germany) using an HC PL APO 63×/1.20 W CS2 motCORR objective, as described by Havurinne et al. (2025).

### Sea slug behaviour under different light spectra

In the feeding and starvation experiments, it was frequently observed that sea slugs showed greater parapodial closure under blue light compared to the other two light spectra. To verify this observation, an additional experiment was conducted to estimate, under the three light treatments, the EDA of the sea slugs (Cartaxana et al., 2019). Sea slugs collected from the maintenance LSS were transferred to a white tray with ASW and allowed to acclimatise under dim room light for 10–15 min. They were then put under red, blue, or white light (the same spectra and PPFD that were used in the feeding and starvation experiments) for 2 min, after which top-down photographs were taken from a fixed position using an EOS 450D digital camera (Canon, Tokyo, Japan). The EDA in mm^2^ was calculated from the photographs with open-source software (ImageJ version 1.54g) (Schneider et al., 2012). To compare the EDA between treatments, the same ten specimens were imaged under white, red, and blue light. Furthermore, additional sea slugs were sequentially exposed to red and blue light, and their behavioural changes were documented using an iPhone 13 Pro Max.

### Statistical analysis

Linear mixed-effects models (LMMs) were used to test significant differences in sea slug fresh weight and photosynthetic activity (Fv/Fm and rETRmax) over time between the different light spectrum treatments. Models were fitted using the ‘lmer’ function from the ‘lme4’ package in R (Bates et al., 2015). The significance of fixed effects was evaluated using the ‘anova’ function provided by the ‘lmerTest’ package in R (Kuznetsova et al., 2017). To assess whether the data met the assumptions of LMMs, the normality of residuals was visually inspected through q–q plots, and the variance heterogeneity was tested using the Breusch–Pagan test from the ‘performance’ package in R (Lüdecke et al., 2021). When necessary, data were transformed with the square root transformation (sqrt) to meet model assumptions. When the assumptions were not met even after the transformation, robust linear mixed models were used through the function ‘rlmer’ from the ‘robustlmm’ package in R (Koller, 2016). Differences in Chl *a* concentration and survival between light treatments were tested with an analysis of variance. Normality and homogeneity of variance were tested using Shapiro–Wilk and Levene's tests, respectively. Tukey's post hoc tests were used to assess for differences between factor levels using the ‘emmeans’ package in R (Lenth, 2025, R package version 1.11.1, https://CRAN.R-project.org/package=emmeans). All statistical analyses were performed using the software R v.4.4.3 (R Core Team, 2025, https://www.r-project.org/).

### Ethics statement

This study was performed in accordance with EU legislation and directives concerning scientific research on animals, including the 3R principles. Ethical approval is not required for studies conducted with non-cephalopod invertebrates. Animals were initially purchased from Tropical Marine Centre (TMC Iberia, Lisbon, Portugal) and reared in the laboratory for several generations. Animals were maintained in LSS in controlled and optimal conditions to ensure maximum fitness.

## Supplementary Material



10.1242/biolopen.062714_sup1 Supplementary information

Dataset 1.
