# Supplementary material for: Red light promotes sea slug growth while blue light increases longevity of stolen chloroplasts
Source: Biol Open. 2026 Sep 28;15(9):bio062714. doi: 10.1242/bio.062714 (PMC13670353; doi:10.1242/bio.062714)
Supplement: Supplementary file 1 [file biolopen-15-062714-s1.pdf]

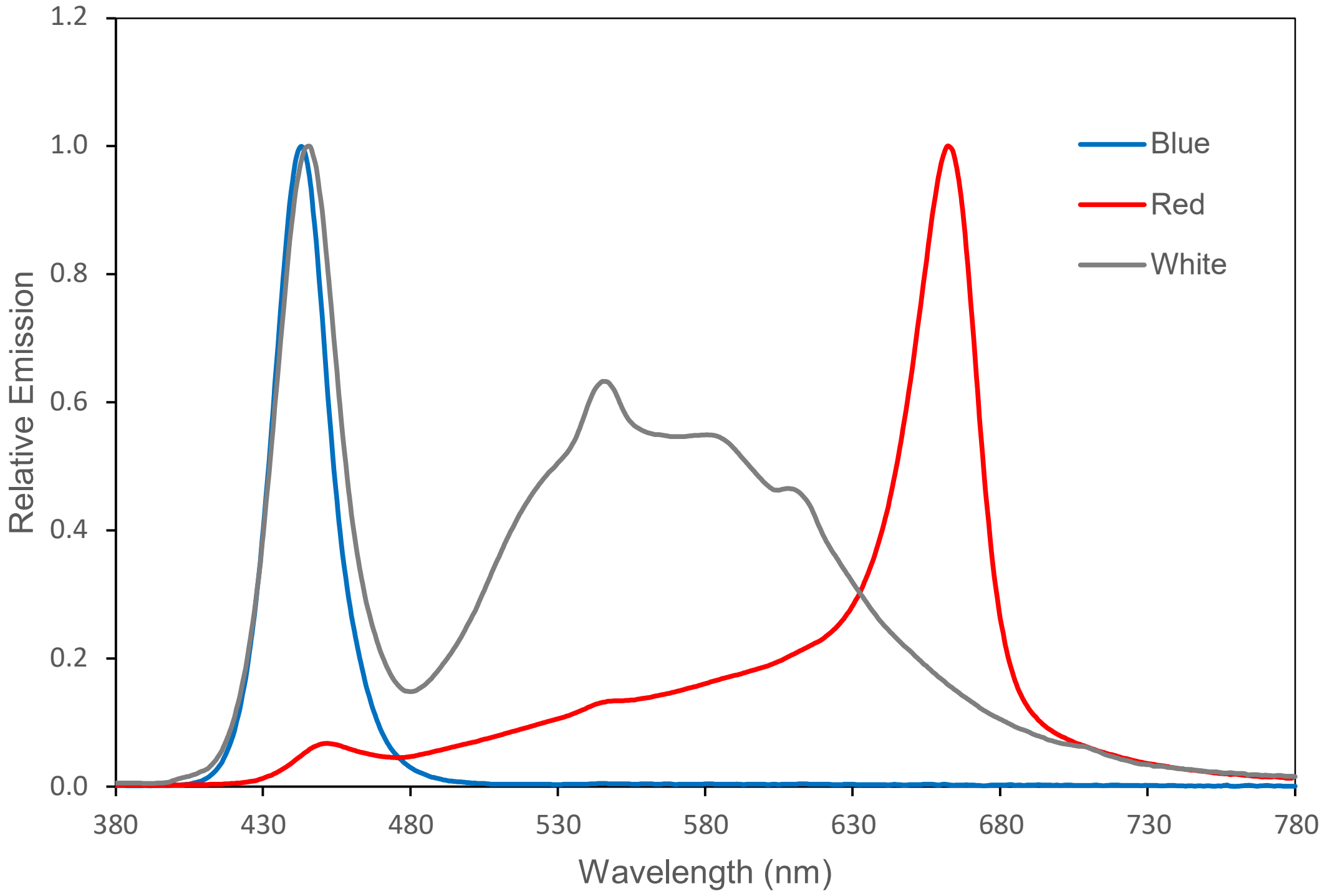

Fig. S1. Emission spectra of the LEDs used in the feeding and starvation experiments of *Elysia crispata*. Spectra are normalized to their maximum values. Photosynthetic photon flux density was, in all cases, 60 µmol photons m^{-2} s^{-1}, measured at the water surface.

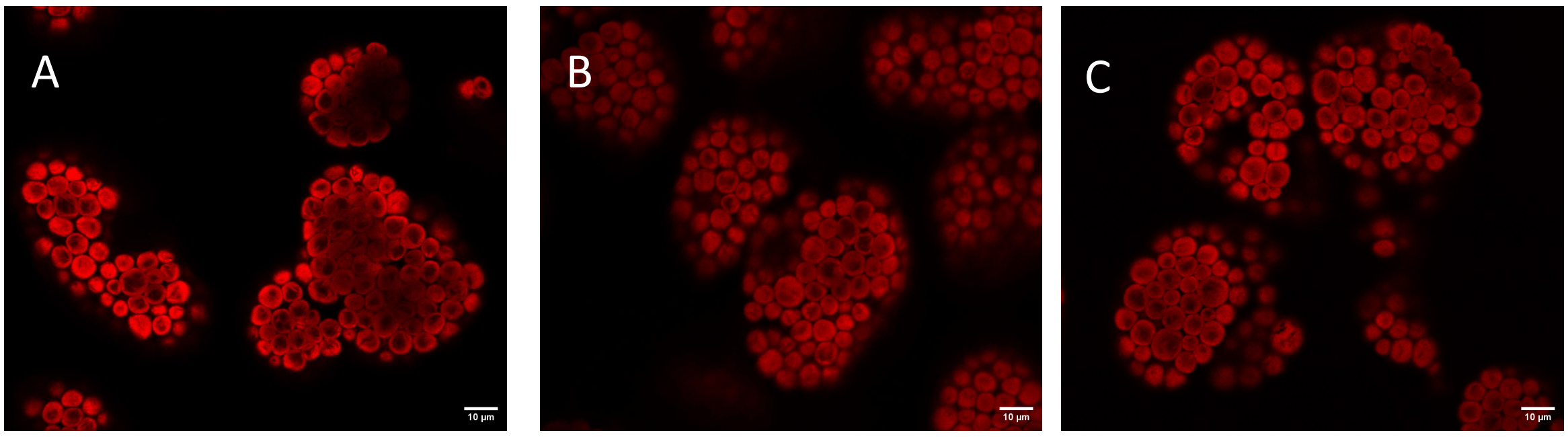

Fig. S2. Representative confocal microscope images of kleptoplasts of *Elysia crispata* reared under different light spectra: white (A), red (B) and blue (C). Sea slugs were maintained at a photosynthetic photon flux density of 60 µmol photons m^{-2} s^{-1} and fed once per week with the macroalga *Bryopsis* sp. for 42 days. Red color in the images corresponds to chlorophyll *a* fluorescence. Scale bar represents 10 µm.

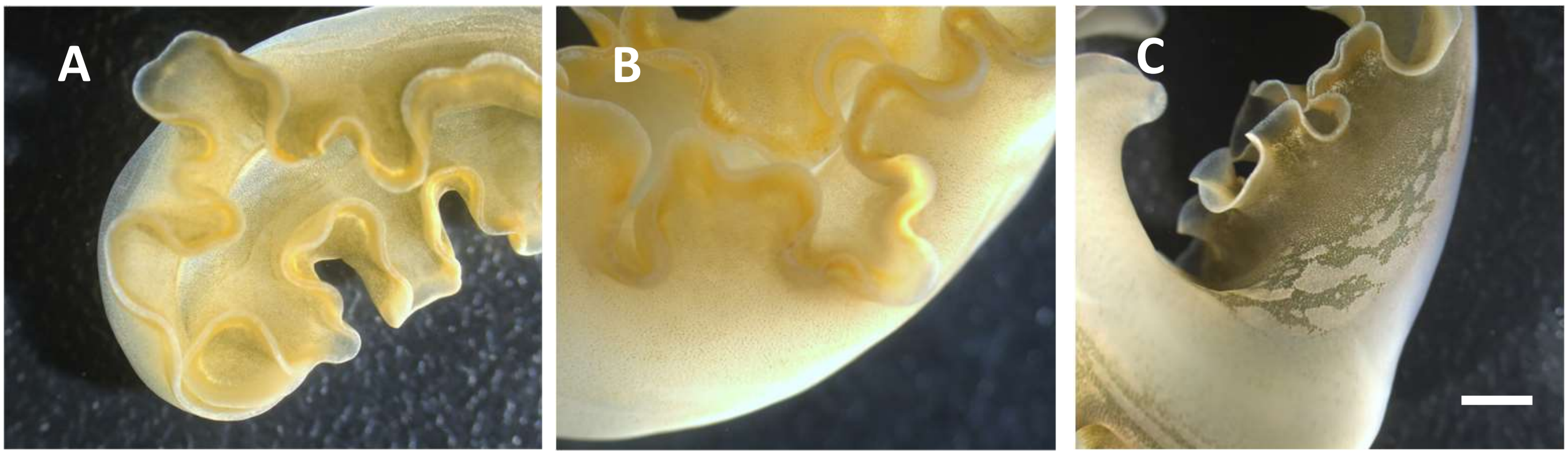

Fig. S3. Details of the parapodia of three representative individuals of *Elysia crispata* after 35 days of starvation reared under different light spectra: white (A), red (B) and blue (C). Sea slugs were maintained at a photosynthetic photon flux density of 60 µmol photons m^{-2} s^{-1}. Photos were obtained with a digital microscope (DMS-300, Leica Microsystems, Wetzlar, Germany). Scale bar represents 2 mm.

Dataset 1.

Available for download at
https://journals.biologists.com/bio/article-lookup/doi/10.1242/bio.062714#supplementary-data

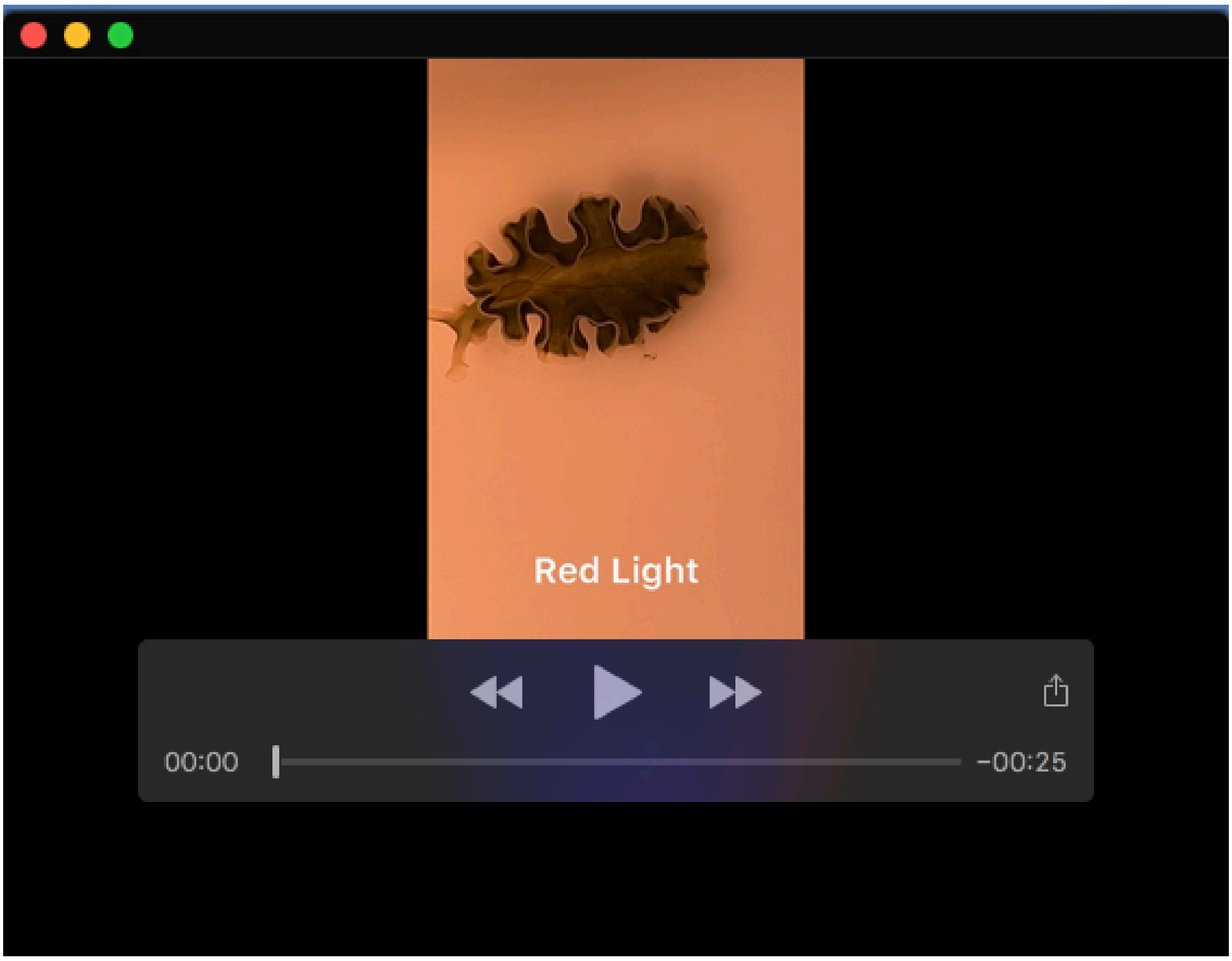

Movie 1. Behavioural response of *Elysia crispata* to different light spectra. Sea slugs were sequentially exposed to a photosynthetic photon flux density of 60 µmol photons m^{-2} s^{-1} of red and blue light. Behavioural changes were documented using an iPhone 13 Pro Max.
