# Supplementary material for: Red light promotes sea slug growth while blue light increases longevity of stolen chloroplasts
Source: Biol Open. 2026 Sep 28;15(9):bio062714. doi: 10.1242/bio.062714 (PMC13670353; doi:10.1242/bio.062714)
Supplement: Supplementary file 2 [file biolopen-15-062714_review_history.pdf]

Red light promotes sea slug growth while blue light increases longevity of stolen chloroplasts

Carlos Paya, Heta Mattila, Vesa Havurinne, Felisa Rey, Sónia Cruz and Paulo Cartaxana
10.1242/bio.062714

Editor: Valentina di Santo

Review timeline

Original submission:	26 May 2026
Editorial decision:	30 June 2026
First revision received:	31 July 2026
Accepted:	4 August 2026

Original submission

First decision letter

MS ID#: bio.062714

MS Title: Red light promotes sea slug growth while blue light increases longevity of stolen chloroplasts

Authors: Carlos Paya, Heta Mattila, Vesa Havurinne, Felisa Rey, Sónia Cruz and Paulo Cartaxana

I have now reached a decision on the above manuscript.

The reviewer reports are shown at the bottom of this email.

Both reviewers found the topic novel and the work well conducted, and I share their enthusiasm for the questions you're addressing. That said, they've raised several points that I'd like you to address before we can move forward, outlined below.

The most important issue concerns interpretation of your light treatments. Without a dark control or a weak-photosynthesis-stimulating condition (such as green light), it's difficult for readers to know what differences between red, blue, and white light actually tell us, particularly given that many of your effect sizes are modest. Relatedly, several of your conclusions attribute differences between light conditions to photosynthesis and kleptoplasty, but this link isn't directly tested in the current experiments. We'd encourage you to consider adding experiments using dark conditions or a photosynthesis inhibitor such as DCMU to support these claims more directly. If that isn't feasible, please revise the text so this remains clearly flagged as an interpretation rather than a demonstrated mechanism, so readers aren't misled.

For the growth experiment, please consider whether the faster growth you observe in red light could simply reflect increased feeding. If possible, accounting for food intake, for example by measuring leftover food, would help rule this out as an alternative explanation.

Both reviewers were intrigued by the parapodial opening and closing behavior you describe, but noted that it isn't quantified. We'd ask that you provide quantitative data here, including how many individuals were observed and how many displayed the described responses, since this behavior may help explain some of your other results.

© 2026. Published by The Company of Biologists under the terms of the Creative Commons Attribution License (https://creativecommons.org/licenses/by/4.0/).

A few smaller points would also strengthen the manuscript. Showing weight loss during starvation normalized to starting mass would make the treatment comparisons easier to interpret given the small effect sizes involved. We'd also ask that you tie the discussion more closely to your specific findings, present the role of photosynthesis in buffering starvation weight loss more cautiously given mixed evidence in the literature (including PMID: 24258718), explain the absence of Day 7 ETR data, and acknowledge that other explanations remain plausible for the chloroplast degradation differences you cite, since this wasn't directly tested.

Finally, the second reviewer raised three points we'd like you to consider. Because the same individuals were used first in the fed condition and then the starvation condition, please discuss the possibility that the starvation results were influenced by the prior feeding experiment. Please also discuss whether the different light conditions may have affected the algal symbionts themselves, independent of slug physiology, as this could be an alternative driver of the patterns you observe. Lastly, please report on the reproductive condition of the animals used, since this can influence weight changes over time.

We think these revisions will meaningfully strengthen an already interesting paper, and we look forward to seeing your revised manuscript. Please don't hesitate to reach out if anything in this letter is unclear.

At this stage, we also ask you to ensure your manuscript complies with our formatting guidelines – please see our manuscript preparation guidelines for details. Provided you are able to fully address the referees' comments, we are positive about publication of your paper (we accept over 95% of revision submissions) and therefore hope you won't mind any extra work involved in reformatting your manuscript at this point.

Please upload both a 'clean' version of your Word file, along with a highlighted version clearly showing where you have made changes in the revised manuscript. Please avoid using 'Track changes' in Word files as these are lost in PDF conversion.

I should be grateful if you would also provide a point-by-point response detailing how you have dealt with the points raised by the reviewers in the 'Response to Reviewers' box. Please attend to all of the reviewers' comments. If you do not agree with any of their criticisms or suggestions please explain clearly why this is so.

Reviewer 1

Comments for the author

This study presents two complementary experiments: (1) feeding slugs under three lighting conditions (white, red, blue), and (2) starving slugs under the same three lighting conditions.

In the first experiment, slugs in red light grow faster than other lighting conditions, despite no difference in rates of photosynthesis as proxied by PAM. The reason for this difference is unclear. Photosynthesis appears to drop and rebound during feeding, but the reason is also unclear. Chloroplasts appear unchanged during feeding experiments.

In the second experiment, slugs starved under each lighting condition lost weight, with the red-light condition declining slowest. The difference appears to be driven by a small gain in mass immediately following starvation in the red-light condition. After 42 days, slugs from all treatments had reached similar reductions in mass. Slugs in blue light appear to retain photosynthetic function assayed by PAM longer than in other lighting conditions.

Major Points

-No measurements are made in the dark, or under lighting conditions that stimulate photosynthesis weakly, such as green. It is hard to interpret what differences between red, blue, and white light might indicate without these comparisons, given all should be expected to stimulate photosynthesis. This seems particularly important given most effect sizes are modest.

© 2026. Published by The Company of Biologists under the terms of the Creative Commons Attribution License (https://creativecommons.org/licenses/by/4.0/).

-In many cases, differences in light conditions are attributed to photosynthesis and kleptoplasty, but this is not tested. Experiments in the dark, using photosynthesis inhibitors (DCMU), or similar are required for this claim. While addition of these experiments would strengthen the conclusions, at minimum this limitation needs to be made clear via text edits to avoid misleading readers.

-For growth experiments, the mostly plausible explanation for slugs in red growing fastest is that they eat more, which seems possible given experimental design. Can this be accounted for, for example by measuring "leftover" food?

-The results of parapodial opening and closing under different light conditions are interesting but not quantified - quantification would significantly strengthen the paper, as the other results seem like they might be at least partially explained by parapodial opening.

Minor Points

-Weight decline during starvation is hard to compare across treatments given small effect sizes and differences in starting mass. Weight during starvation could also be shown normalized starting mass.

-While the introduction does a nice job of summarizing some of the related studies, many discussion points do not seem obviously related to the current study.

-In the introduction, the idea that photosynthesis is relevant for buffering weight loss during starvation in slugs is presented as a given, but some studies (e.g. PMID: 24258718) report the opposite result, and this remains controversial. It seems most fair to say that if photosynthesis does have an effect, it is modest at best.

-Day 7 ETR data was not collected/reported but could be useful to test whether the ETR is higher in red-light exposed slugs vs blue light slugs at that time point.

-Differences in chloroplast degradation are cited as the reason underlying differences between light conditions, but this is not tested. While plausible, other possibilities seem very feasible.

Reviewer 2

Comments for the author

In this paper, the authors report weight and physiological changes of the kleptoplastic sacoglossan Elysia crispata reared under blue, red, or while light with fed and starved conditions.
The topic is novel and interesting, and the work is properly conducted and the conclusions are adequately supported by the data. I have only four comments as listed below.

1. The sea slugs were first reared under the fed condition, then the same individuals were used for the unfed condition. Thus, the results of unfed condition might have been affected by the results of fed condition and should be treated with care. I think some discussion is needed for the possible effects of the previous experiment.

2. Didn't the different light affect the algal condition, which in turn might affect the performance of sea slugs? I think that the authors may want to discuss this possibility in the Discussion.

3. I understand that the sea slugs used had not previously mated or laid eggs, and none of them laid eggs during the experiments. Please refer to the reproductive conditions of the animals used, as these will affect weight changes.

4. In the section "Sea slug behaviour under red and blue light" (line 278-), no quantitative data were presented. If possible, please explain how many individuals did you observe and how many showed the responses explained in the text.

Reviewer's Responses to Questions

© 2026. Published by The Company of Biologists under the terms of the Creative Commons Attribution License (https://creativecommons.org/licenses/by/4.0/).

Experimental quality
Are experiments performed using appropriate methods that will answer the question (or test the hypothesis or support the observations) posed by the authors? Is the right tool used for the job?
If 'No', please indicate reasons in Comments for Author box below.
Reviewer #1:
- Yes

Reviewer #2:
- Yes

Were the data analyzed using appropriate statistical tests?
If 'No', please indicate reasons in Comments for Author box below.
Reviewer #1:
- Yes

Reviewer #2:
- Yes

Reproducibility
Were experiments performed using adequate number of biological replicates?
If 'No', please indicate reasons in Comments for Author box below.
Reviewer #1:
- Yes

Reviewer #2:
- Yes

Is there sufficient raw data to assess the rigor of the analysis?
If 'No', please indicate reasons in Comments for Author box below.
Reviewer #1:
- Yes

Reviewer #2:
- Yes

Does the methods section provide sufficient detail to permit reproducibility?
If 'No', please indicate reasons in Comments for Author box below.
Reviewer #1:
- Yes

Reviewer #2:
- Yes

Completeness
Are the manuscript's conclusions supported by the data?
If 'No', please indicate reasons in Comments for Author box below.
Reviewer #1:
- No

Reviewer #2:
- Yes

Scholarship
Do the authors cite and discuss the merits of data that would argue for and against their conclusion?
If 'No', please indicate reasons in Comments for Author box below.
Reviewer #1:
- Yes

© 2026. Published by The Company of Biologists under the terms of the Creative Commons Attribution License (https://creativecommons.org/licenses/by/4.0/).

Reviewer #2:
- Yes

Does the manuscript title & abstract accurately reflect the contents of the manuscript, without hyperbole?
If 'No', please indicate reasons in Comments for Author box below.
Reviewer #1:
- Yes

Reviewer #2:
- Yes

First revision

Author response to reviewers' comments

Reviewer 1: Summary

This study presents two complementary experiments: (1) feeding slugs under three lighting conditions (white, red, blue), and (2) starving slugs under the same three lighting conditions.

In the first experiment, slugs in red light grow faster than other lighting conditions, despite no difference in rates of photosynthesis as proxied by PAM. The reason for this difference is unclear. Photosynthesis appears to drop and rebound during feeding, but the reason is also unclear. Chloroplasts appear unchanged during feeding experiments.

In the second experiment, slugs starved under each lighting condition lost weight, with the red-light condition declining slowest. The difference appears to be driven by a small gain in mass immediately following starvation in the red-light condition. After 42 days, slugs from all treatments had reached similar reductions in mass. Slugs in blue light appear to retain photosynthetic function assayed by PAM longer than in other lighting conditions.

Major Points

R1#1. No measurements are made in the dark, or under lighting conditions that stimulate photosynthesis weakly, such as green. It is hard to interpret what differences between red, blue, and white light might indicate without these comparisons, given all should be expected to stimulate photosynthesis. This seems particularly important given most effect sizes are modest.

AND

R1#2. In many cases, differences in light conditions are attributed to photosynthesis and kleptoplasty, but this is not tested. Experiments in the dark, using photosynthesis inhibitors (DCMU), or similar are required for this claim. While addition of these experiments would strengthen the conclusions, at minimum this limitation needs to be made clear via text edits to avoid misleading readers.

Reply: This is a very good point. The use of DCMU or a similar herbicide was not possible in this experiment as we use large life-support systems of 340 L of artificial sea water that maximize animal welfare. We know that previous studies, like the one mentioned by the reviewer in another of his comments (Christa et al. 2013; PMID: 24258718) use very small volumes of water and perform experiments using Petri dishes. *Elysia crispata* is a relatively large sea slug that secretes substantial amounts of mucus, which rapidly deteriorates water quality unless a dedicated life-support system is used (for details, see Cartaxana et al. 2026, https://doi.org/10.3390/biology15020168).

© 2026. Published by The Company of Biologists under the terms of the Creative Commons Attribution License (https://creativecommons.org/licenses/by/4.0/).

Additionally, DCMU is not a specific inhibitor of photosynthesis alone, as it may also induce broader physiological and metabolic effects in the host animal. On the other hand, the use of dark conditions can prevent photosynthesis but also affects the animal circadian rhythms and we have observed in a related species (*Elysia viridis*) that it substantially affects feeding on the macroalgae.

Therefore, as suggested, we have revised the text so that differences between light conditions attributed to photosynthesis and kleptoplasty remain clearly flagged as an interpretation rather than a demonstrated mechanism, so readers aren't misled. For example:

Abstract:

“Results suggest a trade-off between increased photosynthetic rates and kleptoplast degradation leading to increased growth (under red light), and enhanced kleptoplast longevity, possibly due to reduced photooxidative damage (under blue light). We hypothesize that the higher degree of parapodia closure observed under blue light shields kleptoplasts from light-induced damage, effectively extending their lifespan”.

Discussion:

“Exposure to red light seems to promote sea slug growth, possibly by transiently increasing photosynthetic rates and earlier kleptoplast degradation. On the other hand, blue light appears to promote closure of the parapodia possibly leading to a lower rate of photooxidative damage, enhancing kleptoplast longevity.”

R1#3. For growth experiments, the mostly plausible explanation for slugs in red growing fastest is that they eat more, which seems possible given experimental design. Can this be accounted for, for example by measuring "leftover" food?

Reply: The “leftover” food was visually inspected and it was clear that the animals were eating all the available food, emptying the cellular content of all the provided algae, within 48 h in all the 3 light treatments. This was also the reason why we increased food availability after week 2 from 100 to 150 mg of *Bryopsis* as detailed in the Materials and Methods. For clarity, we have included a sentence explaining this in the Materials and Methods and discuss this point in the Discussion section:

Materials and Methods

“No differential feeding behaviour was observed between spectral treatments. Colourless algal debris was removed from the wells after 48 h.”

AND

Discussion

“Juveniles of *E. crispata* exhibited sustained weight gain over time under all tested light spectra (white, red, and blue). No apparent differences in the feeding behaviour were observed and the sea slugs emptied the cellular content of the provided food algae within 48 h independently of the light treatment. However, a consistent trend of higher fresh weight under red light was observed. Hence,…”

R1#4. The results of parapodial opening and closing under different light conditions are interesting but not quantified - quantification would significantly strengthen the paper, as the other results seem like they might be at least partially explained by parapodial opening.

Reply: We have executed and additional experiment to quantify the observed behaviour and calculated Exposed Dorsal Area as described in Cartaxana et al. 2019 (https://doi.org/10.1242/jeb.202580). We have added a new Figure 7 to the manuscript and changed Results and Discussion accordingly. The results confirm what was observed in the movie provided.

© 2026. Published by The Company of Biologists under the terms of the Creative Commons Attribution License (https://creativecommons.org/licenses/by/4.0/).

Minor Points

R1#5. Weight decline during starvation is hard to compare across treatments given small effect sizes and differences in starting mass. Weight during starvation could also be shown normalized starting mass.

Reply: We have normalized weight to the starting mass and present a new Figure 4.

R1#6. While the introduction does a nice job of summarizing some of the related studies, many discussion points do not seem obviously related to the current study.

Reply: The Introduction is a small, three-paragraph text that intends to present the subject to non-specialists. In our opinion, it is appropriate, but we are willing to revise it if the reviewer (or the Editor) specifies the points to remove.

R1#7. In the introduction, the idea that photosynthesis is relevant for buffering weight loss during starvation in slugs is presented as a given, but some studies (e.g. PMID: 24258718) report the opposite result, and this remains controversial. It seems most fair to say that if photosynthesis does have an effect, it is modest at best.

Reply: Although, as the reviewer mention, the reference given presents opposite results, I wouldn't refer to this as a controversial issue. There are at least 10 studies reporting the opposite of Christa et al. 2013 (three are cited in the Introduction). Christa et al. 2013 reports comparable weight loss in *Plakobranchus ocellatus* starved under dark and light conditions and in the presence of a photosynthesis inhibitor. However, that study's conclusion that kleptoplast photosynthesis has no impact on the survival of sacoglossan sea slugs during periods of food shortage was clearly overstated, as the authors based it on an extremely low sample size (2 sea slugs per condition). The pitfalls of Christa et al. 2013 have been addressed several times in the literature (for example, see an extended critic of this paper in Pierce et al. 2015, https://doi.org/10.1111/ivb.12082), so we do not think it is appropriate to reintroduce this point once again.

R1#8. Day 7 ETR data was not collected/reported but could be useful to test whether the ETR is higher in red-light exposed slugs vs blue light slugs at that time point.

Reply: We did not collect ETR data at Day 7. The rapid light curves (RLC) involved some manipulation of the animals (as detailed in the Materials and Methods) and a compromise was decided measuring RLC only three and six weeks in the feeding experiment, and after two and four weeks in the starvation experiment, as explained in the Materials and Methods.

R1#9. Differences in chloroplast degradation are cited as the reason underlying differences between light conditions, but this is not tested. While plausible, other possibilities seem very feasible.

Reply: While we did not measure kleptoplast degradation directly, we quantified chlorophyll a as a proxy of the kleptoplast pool and, indirectly, of kleptoplast degradation under the three light treatments. Regardless, we have revised the writing to emphasize that chloroplast degradation was not directly measured.

"In the present work, we report higher Chl *a* concentration (relative to sea slug mass) in fed individuals under blue light than in red and white light. This result suggest that the rate of kleptoplast degradation is impacted by the light spectrum, with blue light enhancing kleptoplast longevity."

Reviewer 2: Review of bio.062714

In this paper, the authors report weight and physiological changes of the kleptoplastic sacoglossan Elysia crispata reared under blue, red, or while light with fed and starved conditions.
The topic is novel and interesting, and the work is properly conducted and the conclusions are adequately supported by the data. I have only four comments as listed below.

© 2026. Published by The Company of Biologists under the terms of the Creative Commons Attribution License (https://creativecommons.org/licenses/by/4.0/).

R2#1. The sea slugs were first reared under the fed condition, then the same individuals were used for the unfed condition. Thus, the results of unfed condition might have been affected by the results of fed condition and should be treated with care. I think some discussion is needed for the possible effects of the previous experiment.

Reply: We are sorry that it was perceived by the reviewer that the same sea slugs were used in the two experiments. This was not the case. This is now clearly stated in the Materials and Methods:

"The animals used in the two experiments (feeding and starvation) were different specimens."

R2#2. Didn't the different light affect the algal condition, which in turn might affect the performance of sea slugs? I think that the authors may want to discuss this possibility in the Discussion.

Reply: The macroalgae offered to the sea slugs were cultured at the same light intensity and at a full light visible spectrum (similar to the white light treatment), as specified in the Materials and Methods, section *Bryopsis* sp. culturing. Since the algae were subjected to different light spectra for a limited period, of up to 48 h, during which they were also being eaten, we believe the effect of the algal condition to be minimal.

R2#3. I understand that the sea slugs used had not previously mated or laid eggs, and none of them laid eggs during the experiments. Please refer to the reproductive conditions of the animals used, as these will affect weight changes.

Reply: No egg masses were spawn in both experiments. In the starvation experiment, although the specimens were sexually mature hermaphrodites, they use cross fertilization exchanging sperm between partners. The sea slugs were individualized, so this was not possible. In the feeding experiment, the juveniles were not only individualized but also not sexually mature. This is now specified in the Materials and Methods.

"In both experiments, sea slugs were placed in separated wells, which prevented them from reproducing by cross-fertilization. No egg masses were spawn during either of the experiments."

R2#4. In the section "Sea slug behaviour under red and blue light" (line 278-), no quantitative data were presented. If possible, please explain how many individuals did you observe and how many showed the responses explained in the text.

Reply: We have executed and additional experiment to quantify the observed behaviour and calculated Exposed Dorsal Area as described in Cartaxana et al. 2019 (https://doi.org/10.1242/jeb.202580). We have added a new Figure 7 to the manuscript and changed Results and Discussion accordingly. The results confirm what was observed in the movie provided.

Second decision letter

MS ID#: bio.062714R1

MS Title: Red light promotes sea slug growth while blue light increases longevity of stolen chloroplasts

Authors: Carlos Paya, Heta Mattila, Vesa Havurinne, Felisa Rey, Sónia Cruz and Paulo Cartaxana

I am happy to tell you that your manuscript has been accepted for publication in Biology Open, pending our standard publication integrity checks. It was accepted on 4th August 2026.

© 2026. Published by The Company of Biologists under the terms of the Creative Commons Attribution License (https://creativecommons.org/licenses/by/4.0/).
